# Phylogeographical Analysis of the Freshwater Gudgeon *Huigobio chenhsienensis* (Cypriniformes: Gobionidae) in Southern China

**DOI:** 10.3390/life12071024

**Published:** 2022-07-09

**Authors:** Xishu Yang, Xiaomin Ni, Cuizhang Fu

**Affiliations:** Ministry of Education Key Laboratory for Biodiversity Science and Ecological Engineering, Coastal Ecosystems Research Station of the Yangtze River Estuary, Institute of Biodiversity Science and Institute of Eco-Chongming, School of Life Sciences, Fudan University, Shanghai 200082, China; yangxishu1990@163.com (X.Y.); 18110700109@fudan.edu.cn (X.N.)

**Keywords:** Cypriniformes, Gobionidae, *Huigobio*, phylogeography, southern China

## Abstract

The freshwater gudgeon *Huigobio chenhsienensis* (Cypriniformes: Gobionidae) is a small fish endemic to southern China. In this study, we used mitochondrial cytochrome *b* gene (*Cytb*), from wide-ranging samplings of *H. chenhsienensis* from the Ou River (the central of southern China) to the Yangtze River Basin (the northernmost part of southern China) to explore genetic variations and the evolutionary history of *H. chenhsienensis* in southern China. In total, 66 haplotypes were identified from *Cytb* sequences of 142 *H. chenhsienensis* individuals, which could be divided into lineages A, B, and C with divergence times of ~4.24 Ma and ~3.03 Ma. Lineage A was distributed in the lower reaches of the Yangtze River, the Oujiang River, and the Jiao River, lineage B was distributed in the Qiantang River and the Cao’e River, whereas lineage C was restricted to the Poyang Lake drainage from the middle reaches of the Yangtze River. Lineage A could be subdivided into sub-lineages A-I, A-II, A-III, and A-IV, with divergence times of 1.30, 0.97, and 0.44 Ma. Lineage C could be subdivided into sub-lineages C-I and C-II, with a divergence time of 0.85 Ma. Our findings indicate that climate change during the Pliocene and Pleistocene eras, as well as the limited dispersal ability of *H. chenhsienensis*, have been major drivers for shaping the phylogeographical patterns of *H. chenhsienensis*.

## 1. Introduction

Phylogeography focuses on the geographic distributions of inter- and intraspecific lineages, as well as the historical processes that have affected their evolution [1,2]. Freshwater fish in contemporary isolated drainages are often the products of historical river evolutions. Therefore, phylogeographical studies of freshwater fish are significant for understanding the historical reorganization or rearrangements of rivers within and among drainages [3,4,5,6]. The genetic diversity observed within a species is a key facet of biodiversity that reflects the evolutionary history and adaptability of a species as a whole, or its constituent populations [7,8].

Grant and Bowen [9] classified four categories of genetic diversity patterns according to levels of polymorphism for haplotypes and nucleotides. They defined high or low haplotype polymorphism (*h*) as *h* ≥ 0.5 or *h* < 0.5, and high or low nucleotide polymorphism (*π*) as *π* ≥ 0.005 or *π* < 0.005. The Yangtze River is the northern boundary for freshwater fish distributed in southern China [10]. Previous studies (Table S1) have shown two categories of genetic diversity for freshwater fish in southern China, i.e., type II (high *h* value and low *π* value) and type IV (high *h* value and high *π* value), as defined by Grant and Bowen [9]. The potential formation mechanism of type II cases is a genetic bottleneck followed by rapid population expansion [9]. However, widespread climate oscillations in the Pleistocene and secondary contacts resulting from population expansions after the glacial period may have shaped the type IV genetic diversity pattern [11].

Based on the geographical distribution of lineages and the degree of genetic differentiation, Avise [12] summarized five categories of phylogeographic patterns as follows: “Deep gene tree, major lineages allopatric” (pattern I), “Deep gene tree, major lineages broadly sympatric” (pattern II), “Shallow gene tree, lineages allopatric” (pattern III), “Shallow gene tree, lineages sympatric” (pattern IV), and “Shallow gene tree, lineage distributions varied” (pattern V). Prior studies on freshwater fish in southern China (Table S2) have shown three categories of phylogeographic patterns. The main driver of pattern I is the long-term isolation of drainages resulting from biogeographic barriers formed by geological tectonic movement [13,14,15]. The main driver of pattern II is river capture or historical connections of the river facilitated by sea level fall during the Pleistocene glacial period [16,17]. Pattern V is relatively rare in nature, and is attributed to the low contemporary gene flow between populations which have historically been closely connected [12,18]. Although Chinese ichthyologists have carried out phylogeographical studies on a variety of fish in southern drainages, it is still necessary to add phylogeographical cases of fish species in the above area so that more evidence can be accumulated to understand the evolutionary processes that generate and maintain intraspecific genetic variation, and lay a scientific foundation for the effective management and rational protection of genetic resources for freshwater fish in China.

*Huigobio chenhsienensis* Fang 1938 is classified under the order Cypriniformes, family Gobionidae, subfamily Gobioninae, and genus *Huigobio* [19,20]. It is a species endemic to southern China, and is distributed from the Ou River to the Yangtze River [20,21,22,23]. *H. chenhsienensis* is small (standard length of less than 8 cm) and usually inhabits the upper reaches of streams, feeding mainly on benthic algae [21,22,24]. The small body size and stream headwater habitats may indicate the potential limited dispersal ability of *H. chenhsienensis*. In addition to *H. chenhsienensis*, the genus *Huigobio* comprises two other species: *H. exilicauda* Jiang and Zhang 2013 and *H. heterocheilus* Sun, Li, Tang and Zhao 2022. *H. exilicauda* is distributed in the Pearl River, whereas *H. heterocheilus* is found in the Xiang River, a tributary of Dongting Lake drainage in the middle reaches of the Yangtze River [22,23].

In this study, mitochondrial cytochrome *b* gene (*Cytb*) sequences were obtained from samples covering the distribution drainages of *H. chenhsienensis*. Based on analyses of phylogeny, genetic structure, and population history, we discuss the roles of river evolution and paleoclimatic events in the formation of phylogeographic patterns of *H. chenhsienensis* in southern China.

## 2. Materials and Methods

### 2.1. Sample Collection

**Figure 1:**
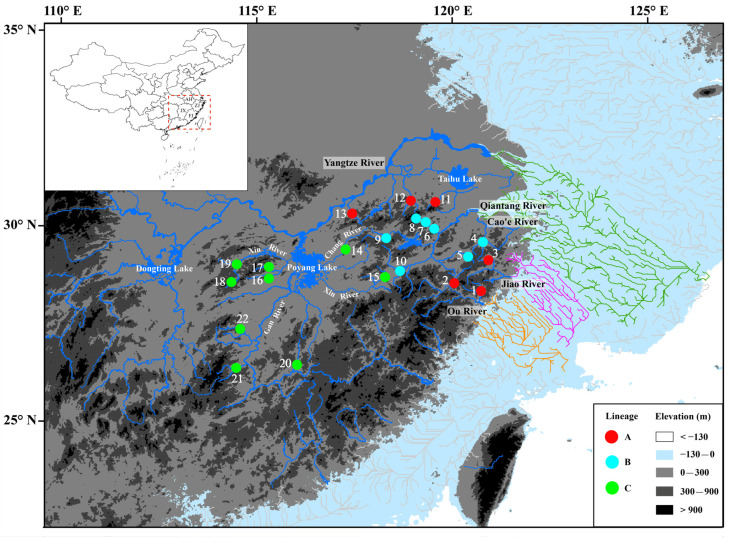
Localities of *Huigobio chenhsienensis* specimens and spatial distributions of mitochondrial cytochrome *b* gene (*Cytb*) lineages. The green (paleo-Yangtze River), magenta (paleo-Jiao River), and orange (paleo-Ou River) lines represent paleo-channels of coastal rivers’ downward movement to continental shelf at an elevation of −130–0 m during the last glacial maximum (LGM) when the sea level fell by about 130 m. The code of each population is shown in Table 1 and lineages are defined in Figure 2. FJ—Fujian Province; JX—Jiangxi Province; ZJ—Zhejiang Province; AH—Anhui Province.

From November 2011 to September 2019, 142 specimens of *H. chenhsienensis* were collected from the Ou River, Jiao River, Qiantang River, Cao’e River, and Yangtze River, using small set nets and gill nets by ourselves or with help from local fishermen. There are a total of 22 localities, with 1 to 18 specimens for each locality (Figure 1 and Table 1). In order to take into account fish euthanasia, the experimental fish were anesthetized to lose consciousness using the Eugenol. First, the Eugenol (purity ≥ 99.99%) was diluted into an aqueous solution with a concentration of 0.25 mL·L^−1^. Second, experimental fish were placed in the aqueous solution of the Eugenol until they were anesthetized to lose consciousness. Third, the anesthetized fish were fixed in 75% and then transferred to 95% ethanol for long-term storage. All specimens collected in this study were deposited in the Zoological Museum of Fudan University.

### 2.2. Sequence Obtaining and Processing

Genomic DNA was extracted from muscle tissue using a salting-out extraction method [25]. A pair of primers (CGluF: 5′-TCTTGCTCGGAYTTTAACCGAG-3′, CThrR: 5′-TCTTGCTCGGAYTTTAACCGAG-3′) [26] were used for PCR amplification of *Cytb* gene. The PCR amplification was conducted in a 30 μL reaction volume which contained 1.2 μL of template DNA, 0.6 μL of each primers, 15.0 μL of 2 × Es Taq MasterMix, and 12.6 μL of ddH_2_O. The reaction conditions of PCR amplification for *Cytb* gene were the same as Chai and Fu [27] except that the primers were annealed at 58 °C. Moreover, the primers and annealing temperature for PCR amplification of the mitochondrial genome used in this study are listed in Table S3. The PCR products were subsequently purified and sequenced by Jieli Biology Co., Ltd., Shanghai, China.

The obtained sequences were assembled in Sequencher v5.4 (Gene Codes, Ann Arbor, MI, USA) and supplemented with manual correction for incorrect base recognition of the chromatograms. Sequence alignment was performed with the default algorithm under MAFFT v7.427 [28]. Amino acid translation was performed in DAMBE v7.2.43 to check whether there were terminators in the sequences [29]. The newly generated *Cytb* sequences were imported into DnaSP v6.12.01 to obtain haplotype data [30]. Basic information on the *Cytb* sequence was acquired using MEGA (Molecular Evolutionary Genetics Analysis) version 7.0.26 (Arizona State University, Philadelphia, PA, USA) [31].

### 2.3. Phylogeographic Analysis and Divergence Time Estimation

The *Cytb* time tree of *H. chenhsienensis* was reconstructed in BEAST v2.6.6 software (University of Auckland, Auckland, New Zealand) [32] based on haplotype sequences. Taking full account of the lack of fossil information on *Huigobio* for estimating the differentiation time of *H. chenhsienensis*, a mitochondrial genome time tree of *Huigobio* and its close relatives was thus developed to obtain the differentiation time of *H. chenhsienensis* and *H. heterocheilus* as the secondary calibration point by using two fossil species of Gobionidae with complete skeletons and corresponding stratigraphic age information (data sources and results are shown in Table S4 and Figure S1). The best base substitution model for the 13 mitochondrial protein genes was obtained based on the automatic search function of the bModelTest module in BEAST v2.6.6, the Birth–Death model was selected, and a tree model was set at the same time [33]. The first fossil species corresponded to the divergence node of Gobionidae and Acheilognathidae [34]. This fossil species, which occurred in the Middle Eocene (50.5–42.0 Ma) [35] is †*Palaeogobio zhongyuanensi* [36], i.e., calibration point 1 (C1), and was set to LogNormal distribution, μ = 3.82, σ = 0.05. The second fossil species corresponded to the divergence node of *Gnathopogon* and *Coreoleuciscus* [37]. This fossil species occurred in the Miocene (20.4–15.0 Ma) [38] and is †*Gnathopogon macrocephala* [36], i.e., calibration point 2 (C2), and was set to LogNormal distribution, μ = 2.86, σ = 0.078. The molecular clock was set as a relaxed clock. Two independent runs of 400 million generations with sampling every 10,000 generations in each run were performed in the Bayesian analysis. The two runs were combined and the first 30% sampling trees were discarded as burn-in. Effective sample size (ESS) was evaluated using Tracer v1.7.0 [39]. When the ESS was greater than 200 for each parameter, the results were considered to be convergent.

To test for the geographic correspondence of haplotypes across all populations of *H. chenhsienensis*, the median-joining network was constructed under NETWORK v10.2.0.0 (Fluxus Technology Ltd., Colchester, England; https://www.fluxus-engineering.com, accessed on 15 January 2022). Combined with the geographical distribution and individual number of haplotypes, it was further modified in Adobe Illustrator.

The interspecific divergence time between *H. chenhsienensis* and *H. heterocheilus* was 11.66 Ma (95% HDP: 13.04–10.44 Ma), which was obtained from the time tree of the mitochondrial genome presented above and used as a secondary calibration point to infer the *Cytb* time tree of *H. chenhsienensis*. The specific settings were as follows: normal distribution, *m* = 11.74, *s* = 0.66. Using the likelihood-ratio test in DAMBE v7.2.43, the result of the molecular clock test (X^2^ = 96.11, d.f. = 69, *p* = 0.0172) did not support the molecular clock model [29], and the relaxed-clock model was set. *H. heterocheilus* was selected as an outgroup. The Birth–Death model was selected as a tree prior. Two independent runs of 100 million generations, with sampling every 1000 generations, were performed. The remaining settings were the same as the time tree construction above.

### 2.4. Genetic Structure and Population History

The genetic diversity of *H. chenhsienensis* was estimated, and the genetic differentiation coefficients, N_ST_ and G_ST_, of the populations were calculated and compared with determination of the presence of a significant genetic structure using DnaSP v6.12.01 [30,40]. The total Φ_ST_ and pairwise comparison Φ_ST_ values among basins were obtained in Arlequin v3.5.1 [41]. To detect the spatial genetic structure of *H. chenhsienensis* populations, the maximum Φ_CT_ value and its corresponding grouping were obtained by simulating the groupings of different populations using SAMOVA v2.0 [42]. For each lineage, Arlequin v3.5.1 was applied to conduct neutrality tests [43,44] and mismatch distribution tests with a spatial expansion model [45]. Bayesian skyline plot analysis of each lineage was implemented in BEAST v2.6.6 [32], the Relaxed Clock Log-Normal distribution model was selected, and the tree model was set as the Coalescent Bayesian Skyline. The best base substitution model used for the analysis was obtained from jModeltest v2 [46]. The *Cytb* mutation rate (1.30% substitutions per site per million years) obtained from the time tree of *Cytb* haplotypes of *H. chenhsienensis* was used in the reconstruction of the Bayesian skyline plot.

### 2.5. Ancestral Area Reconstruction

The ancestral area reconstruction of *H. chenhsienensis* was performed by loading the BioGeoBEARS package version 0.2.1 [47] in the R v3.5.0 program. The analysis used the *Cytb* haplotypes time-calibrated tree of *H. chenhsienensis* as the input tree and the basins as the geographic distribution unit. Finally, the optimal model was selected according to Akaike information criterion (AIC) to reconstruct the ancestral area of *H. chenhsienensis* [48].

### 2.6. Inferring Palaeodrainage along the Coast

Topographic and bathymetric information on a 30 arc-second interval grid at a global scale, with accuracy in meters, was retrieved from the digital elevation model (DEM) of the General Bathymetric Charge of the Oceans 2021 Grid (GEBCO_2021 Grid; http://www.gebco.net/, accessed on 10 November 2021). The maximum sea level decline during the glacial period of the Late Pleistocene was about −130 m [49,50]. Therefore, the elevation and bathymetry data of the maximum sea level retreat during the glacial period of the Late Pleistocene could be used to infer potential connections between palaeodrainages and current basins along the coast of China through the Arc Hydro Tools Box in ArcGIS v.10. The palaeodrainage reconstruction methods were performed in reference to Thomaz et al. [51] with the following steps: first, the DEM of GEBCO_2021 Grid was imported into ArcGIS v.10; secondly, the −130 m basic contours were drawn using the Contour List tool in the Surface Tools box to estimate the maximum extent of continental shelf exposure during the Pleistocene, followed by the generation of a clipped digital elevation model grid chart using the Fill, Flow Direction, Watershed, Flow Accumulation, and Stream Order tools in the Arc Hydro Tools Box. Finally, the raster streams were converted to vectors, and a visualized palaeodrainage reconstruction map was generated using the Stream to Feature tool.

## 3. Results

### 3.1. Phylogenetic Relationships and Divergence Time

**Figure 2:**
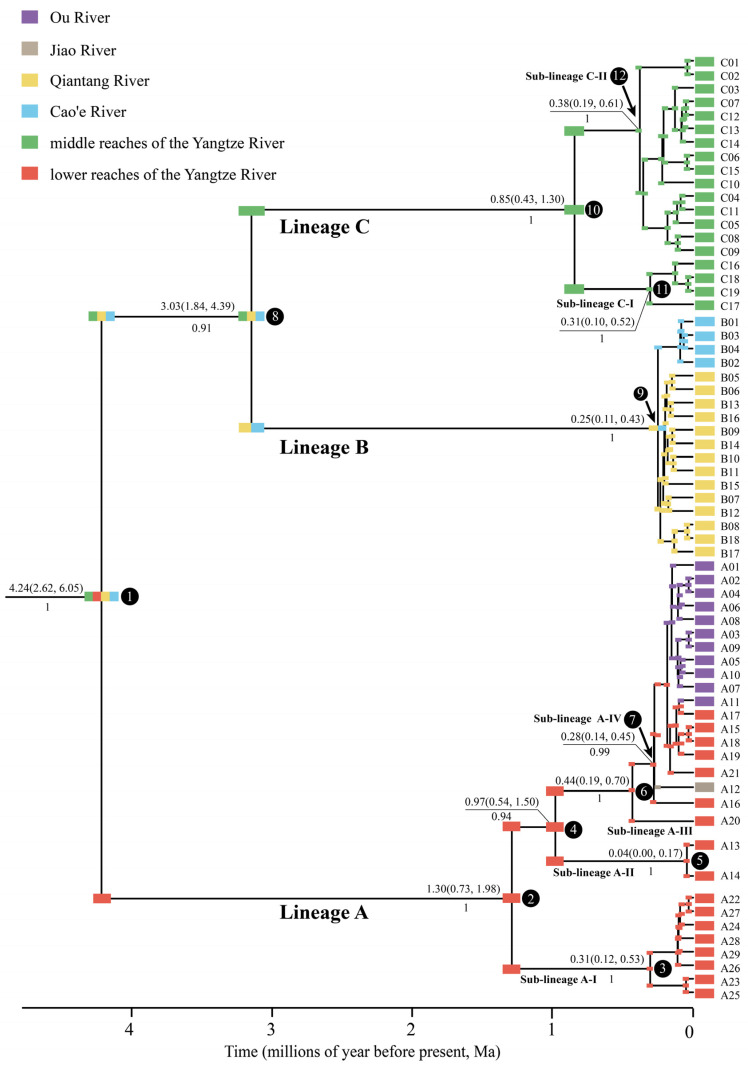
A time-calibrated phylogeny and ancestral area reconstructions among 66 *Cytb* haplotypes of *H. chenhsienensis*. The mean value of the divergence times and their 95% confidence interval are shown above the nodes. The posterior probabilities are below the nodes. The numbers labelled in black circles represent the serial numbers of the major nodes (posterior probability more than 0.9). The outgroup taxon (*Huigobio heterocheilus*) is not shown, and its information in detail is provided in Table S4. The color block at each node reflects ancestral distribution.

A total of 66 haplotypes (Table 1, GenBank Accession: OM647979-OM648044) were detected among the *Cytb* sequences of 142 *H. chenhsienensis* specimens. All the *Cytb* sequences had an identical length (1140 bp), with a GC content of 41.3%, containing 184 variable sites and 161 parsimony informative sites. The time-calibrated phylogeny (Figure 2) showed that 66 *Cytb* haplotypes of *H. chenhsienensis* could be divided into lineages A (29 haplotypes), B (18 haplotypes), and C (19 haplotypes); divergence times among them were 4.24 Ma and 3.03 Ma. The lineage A could be subdivided into four sub-lineages, namely, A-I, A-II, A-III, and A-IV, which diverged from each other at 1.30, 0.97, and 0.44 Ma. The lineage C consisted of two sub-lineages, C-I and C-II, with a divergence time of 0.85 Ma.

**Figure 3:**
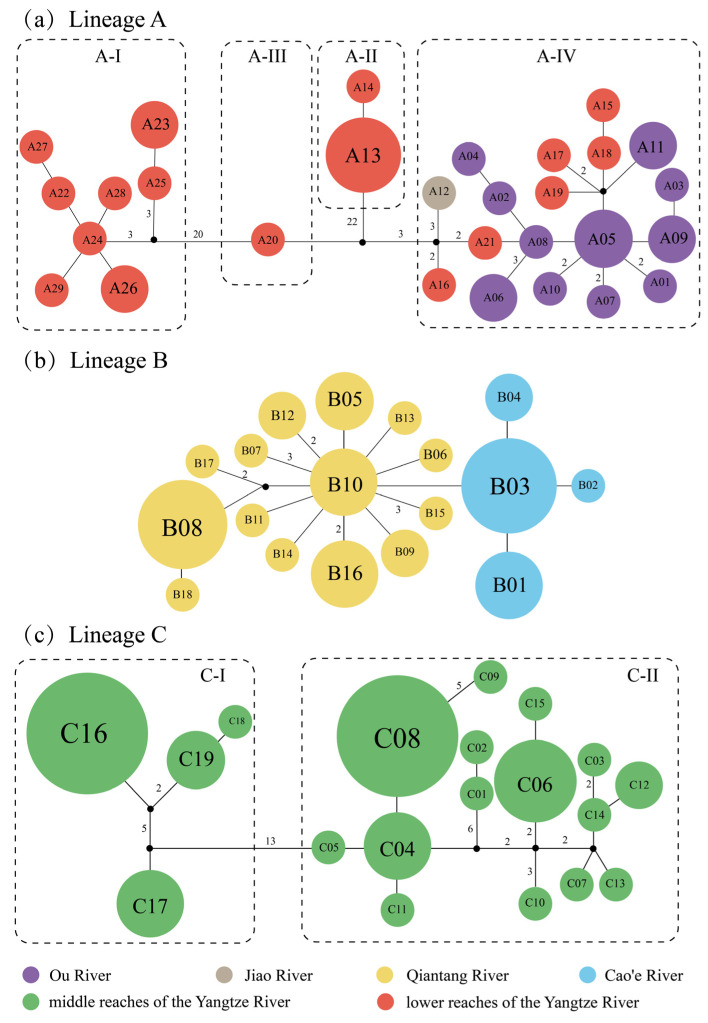
A haplotype network of lineages A (**a**), B (**b**) and C (**c**) for *H. chenhsienensis*. Missing haplotypes are represented with black dots. The number of mutational steps is marked between the haplotypes unless that number is one.

There were 61 mutational steps between lineages A and B + C and 54 mutational steps between lineages B and C observed in the haplotype network. All 66 haplotypes were private haplotypes for each river basin (Figure 3). In lineage A, the three sub-lineages, A-I, A-II, and A-III, were only distributed in the lower reaches of the Yangtze River, whereas the sub-lineage A-IV was distributed across the lower reaches of the Yangtze River, Ou River, and Jiao River (Figure 3a). The lineage B was distributed in the Qiantang River and Cao’e River (Figure 3b). The lineage C was only distributed in Poyang Lake of the middle reaches of the Yangtze River (Figure 1 and Figure 3c), among which the sub-lineage C-I was distributed in Gan River of the Poyang Lake river system (Locality No. 20–22, as shown in Table 1 and Figure 1) and the sub-lineage C-II was distributed in Rao River, Xin River, and Xiu River of the Poyang Lake river system (Locality No. 14–19, as shown in Table 1 and Figure 1).

### 3.2. Genetic Structure

Among the distribution basins of *H. chenhsienensis* (excluding Jiao River with one specimen), haplotype diversity (Table 2) was highest in the Ou River (*h* = 0.9500) and lowest in Cao’e River (*h* = 0.6667); nucleotide diversity (Table 2) was highest in the Yangtze River (*π* = 0.0371) and lowest in Cao’e River (*π* = 0.0007).

Comparison between two genetic differentiation coefficients (N_ST_ > G_ST_: N_ST_ = 0.796, G_ST_ = 0.072) indicated a significant geographic structure for *H. chenhsienensis*. The total Φ_ST_ among basins was 0.627 (*p* = 0.000), and the pairwise comparison Φ_ST_ values among basins were relatively high and significant (Table 3). The SAMOVA divided the five basins into four genetic groups as follows: Yangtze River, Qiantang River, Cao’e River, Ou River + Jiao River, where Φ_CT_ value (=0.741) was the largest.

### 3.3. Demographic History

**Figure 4:**
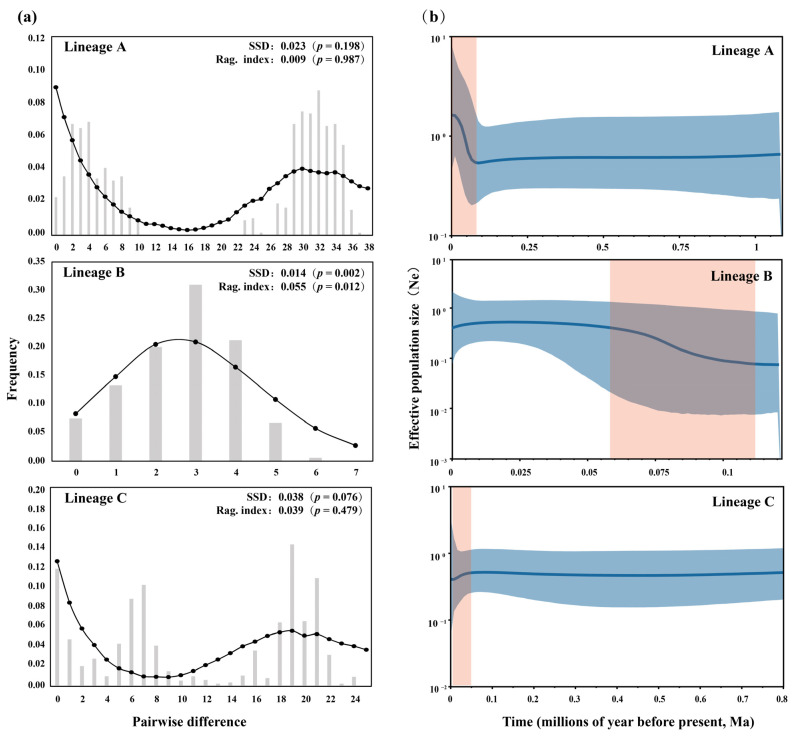
Population history including mismatch distributions (**a**) and Bayesian skyline plots (**b**) for lineages A, B, and C of *H. chenhsienensis*. In the mismatch distributions, the bar graphs represent observed values, and the black solid dots and solid lines represent expected values under the population expansion model. For the Bayesian skyline plots, the actual value of effective population size is represented with the solid blue line, 95% confidence interval is represented with the shaded range, and the time range of population expansion or recession is shown with the red box.

Tajima’s D value and Fu’s Fs value of lineage A for *H. chenhsienensis* were insignificantly positive and marginally significantly negative, respectively, and both indices were significantly negative in lineage B and insignificantly positive in lineage C (Table 4). Bimodal distributions were observed in lineages A and C, whereas lineage B exhibited a unimodal peak (Figure 4a). The Bayesian skyline plot analysis (Figure 4b) revealed that the population expansion of lineages A and B started at 0.088 Ma and 0.110 Ma, respectively; however, lineage C contracted from 0.040 Ma.

### 3.4. Ancestral Area Estimation

By evaluating six biogeographic models of BioGeoBEARS analyses, the DIVALIKE+J model provided an optimal fit to the data based on AIC scores (Table S5). The ancestral area reconstructions based on the optimal model supported the suggestion that the common ancestor of *H. chenhsienensis* was widely distributed in the middle reaches of the Yangtze River, the lower reaches of the Yangtze River, the Qiantang River, and the Cao’e River at 4.24 Ma (95% HPD: 6.05–2.62 Ma). The current geographic distribution pattern was formed after three vicariant and three dispersal events (Figure 2).

### 3.5. Palaeodrainage Reconstructions

Among the distribution basin of *H. chenhsienensis*, the reconstruction of the coastal palaeodrainages (Figure 1) showed that the Yangtze River, Qiantang River, and Cao’e River were interconnected on the exposed continental shelf during the Last Glacial Maximum, whereas the Ou River and Jiao River entered the sea independently during the same period.

## 4. Discussion

### 4.1. Phylogeographic Pattern and Processes

In this study, the lineages A, B, and C of *H. chenhsienensis* were separated from each other over geographic space (Figure 1), and these three lineages were linked by 54 or 61 mutational steps, demonstrating deep genetic differentiation. Thus, the phylogeographic pattern of *H. chenhsienensis* conformed to category I defined by Avise [12], viz., allopatric lineages with deep differentiation. East Asian monsoon studies in geology indicated that the East Asian summer monsoon intensified at about 5.0–4.2 Ma [52,53,54] and 3.7–2.9 Ma [55,56] during the Pliocene (5.333–2.58 Ma), accompanied by noticeably increased temperature and monsoon rainfall. These two intensification phases of the East Asian summer monsoon in the Pliocene coincided well with the divergence times of three lineages for *H. chenhsienensis* (~4.24 Ma, node 1 in Figure 2; ~3.03 Ma, node 8 in Figure 2). At the same time, the global temperature was roughly 2–4 °C warmer in the early mid-Pliocene (ca. 5–3 Ma) than at present [57], which resulted in the global sea level being approximately 10–20 m higher than today [58], suggesting that the basins within distribution regions of *H. chenhsienensis* were isolated from each other during that period. However, *H. chenhsienensis* from each basin did not form a monophyletic group (Figure 1). Hence, two noticeable climatic change events in the early mid-Pliocene may be responsible for the divergence of lineages A, B, and C and the formation of geographical patterns of *H. chenhsienensis*.

The lineage A of *H. chenhsienensis* consisted of four sub-lineages. Among them, three sub-lineages, A-I, A-II, and A-III, were only distributed in the lower reaches of the Yangtze River, whereas the sub-lineage A-IV was distributed sporadically in the Ou River, Jiao River, and the lower reaches of the Yangtze River (Figure 3a). Paleoclimate studies of East Asia showed that the East Asian summer monsoon weakened and the climate became cold and dry at about 1.8–1.2 Ma and 0.9–0.3 Ma during the Pleistocene (2.58–0.0117 Ma), whereas the East Asian summer monsoon strengthened and the climate turned warm and wet at 1.2–0.9 Ma [59]. In addition, all 29 haplotypes of the lineage A were private to each sampling site, implicating the low dispersal ability of *H. chenhsienensis*, inhabiting the upper stream. The geographical distribution and the divergence time (1.30, 0.97, and 0.44 Ma; nodes 2, 4, and 6 in Figure 2, respectively) of the four sub-lineages of lineage A in *H. chenhsienensis* suggested that the low dispersal ability of this species, rather than climate change in the Pleistocene, may be the main driving force of sub-lineage splits and geographical pattern formation of lineage A. Combining phylogenetic relationships of the four sub-lineages of lineage A (Figure 2) and phylogenetic nesting of haplotypes from the Ou River and the Jiao River within haplotypes from the lower reaches of the Yangtze River (Figure 3a), it was suggested that the lineage A originating from the lower reaches of the Yangtze River and *H. chenhsienensis* from the Ou River and Jiao River in the sub-lineage A-IV came from the dispersion of the lower reaches of the Yangtze River population. The result of reconstructing palaeodrainages in this study indicated that the Yangtze River, Ou River, and Jiao River met the sea independently during the Pleistocene glacial period, implying that these three basins were unconnected when the sea level dropped at that time. The time of most recent common ancestor of sub-lineage A-IV was 0.28 Ma (node 7 in Figure 2), during which the global sea level dropped about 60 m [60], but the climate was characterized by warmth and humidity [59,61]. Previous studies have shown that seasonal flooding could facilitate the dispersion of fish through a temporary connection between rivers that meet the sea independently [62,63]. Consequently, the seasonal floods during the late Middle Pleistocene may be the reason for the colonization of *H. chenhsienensis* from the lower reaches of the Yangtze River to the Ou River and Jiao River.

The lineage B of *H. chenhsienensis* was distributed in the Qiantang River and Cao’e River (Figure 1). The 18 haplotypes of this lineage were all restricted to each basin, and the haplotypes in the two basins were linked by only a single mutational step (Figure 3b), which revealed a recent historical connection between the Qiantang River and Cao’e River. The TMRCA of lineage B was 0.25 Ma (node 9 in Figure 2), which falls into the late mid-Pleistocene glacial period, and the global sea level dropped about 80 m at that time [60]. Palaeodrainage reconstruction in this study suggested that the Qiantang River and Cao’e River could be interconnected through palaeodrainages as the sea level dropped during the glacial period (Figure 1). Therefore, a sea level fall during the late mid-Pleistocene glacial period prompted the historical connection between the Qiantang River and Cao’e River, and then drove a close relationship between haplotypes of the two basins from lineage B of *H. chenhsienensis*.

The lineage C of *H. chenhsienensis* comprises two sub-lineages. The sub-lineage C-I was distributed in the Gan River of Poyang Lake drainage (sampling site 20–22), whereas the sub-lineage C-II was distributed in the Rao River (sampling site 14), Xin River (sampling site 15), and Xiu River (sampling site 16–19) of the Poyang Lake drainage. All 19 haplotypes from the two sub-lineages were private haplotypes for each river in the Poyang Lake drainage (Table 1), which implies low gene flow among rivers for the lineage C of *H. chenhsienensis* inhabiting the Poyang Lake drainage. The divergence time between sub-lineages C-I and C-II was 0.85 Ma (node 10 in Figure 2), when the climate of East Asia was cold and dry [59,64]. Consequently, climatic change in the late Early Pleistocene and the low dispersal ability of *H. chenhsienensis* may have driven the lineage divergence and geographic pattern of lineage C.

### 4.2. Genetic Structure and Historical Demography

The fixation coefficient of genetic differentiation, N_ST_, was much higher than G_ST_, indicating that the geographical environment was closely related to lineage differentiation in *H. chenhsienensis*. Among basins, high levels of genetic variation detected in pairwise comparison and lack of shared haplotypes reflected limited gene flow in *H. chenhsienensis*. A high genetic differentiation of fish populations from different basins was also observed in the Yangtze River and its southern drainage systems, for example, *Aphyocypris normalis* [14] and *Hemibagrus guttatus* [65]. An increasing number of cases suggested that smaller freshwater fish inhabiting the middle and lower water column have poor dispersal ability, which is apt to display genetic differentiation, thus resulting in a pronounced genetic structure [66,67,68,69,70]. Therefore, the spatial niche for *H. chenhsienensis* which inhabits the upper reaches of streams and the low dispersal ability owing to its small size (<8 cm) may be responsible for the strong spatial genetic structure of *H. chenhsienensis*.

In this study, the pattern of genetic diversity of *H. chenhsienensis* in the Yangtze River Basin showed high haplotype polymorphism (*h*) and high nucleotide polymorphism (*π*), which may be ascribed to the simultaneous occurrence of lineages A and C. However, there was only a single lineage (A or B) in the Ou River, Qiantang River, or Cao’e River, and the pattern of genetic diversity of *H. chenhsienensis* in each basin was observed as having high *h* values and low *π* values, which suggested that *H. chenhsienensis* populations in these basins may have suffered from genetic bottlenecks, followed by rapid population expansions [9]. This speculation was further supported by the borderline significant or significantly negative Fu’s *Fs* values of lineages A and B, respectively, as well as the unimodal peak in mismatch distribution of the lineage B. Moreover, we detected that the population expansions of lineages A and B started at ~0.088 Ma or ~0.110 Ma, respectively, but the lineage C experienced population contraction from ~0.040 Ma to the present (Figure 4b). Paleoclimate studies of East Asia demonstrated that from 0.110 Ma to 0.040 Ma, there was an interglacial to glacial transition stage in the Late Pleistocene, when the global sea level was 40–50 m lower than the present day, and the climate was cold and dry [71]. Therefore, this study concluded that the cross-basin spatial distribution of lineages A and B of *H. chenhsienensis* was the reason for rapid population growth after the lower-temperature period and reduced rainfall during the Late Pleistocene, whereas the spatial distribution of the lineage C was confined to the Poyang Lake drainage system, causing a severe population bottleneck after the experience of the dry and cold climate in the Late Pleistocene; thus, the population of lineage C had hardly recovered and is in a state of contraction.

## 5. Conclusions

In summary, *H. chenhsienensis* was composed of three lineages, exhibiting a pattern of allopatric lineages with deep divergence. The climate changes during the Pliocene and Pleistocene and the poor dispersal ability of *H. chenhsienensis* might be the major driving forces of its phylogeographical pattern. The Yangtze River population of *H. chenhsienensis* denoted high haplotype polymorphism (*h*) and high nucleotide polymorphism (*π*); other drainage populations exhibited high h and low *π*. During the Late Pleistocene, lineages A and B experienced population expansion, whereas lineage C suffered from severe bottleneck effects. Based on the spatial subdivision of genetic structure for *H. chenhsienensis*, it is suggested that the four conservation geographic units (Yangtze River, Qiantang River, Cao’e River, Jiao River + Ou River) should be classified for management.

## Figures and Tables

**Table 1:** Information on localities of specimens and mitochondrial cytochrome *b* gene (*Cytb*) haplotypes for *Huigobio chenhsiensis*.

Basin and Locality	Lat.	Long.	N	*Cytb* Haplotype
Ou River				
1. Yongjia County, Zhejiang Province	28.35° N	120.75° E	14	A01(1), A02(1), A03(1), A04(1), A05(3), A06(2), A07(1), A08(1), A09(2), A10(1)
2. Jinyun County, Zhejiang Province	28.66° N	120.09° E	2	A11(2)
Jiao River				
3. Tiantai County, Zhejiang Province	29.14° N	121.03° E	1	A12(1)
Cao’e River				
4. Xinchang County, Zhejiang Province	29.51° N	120.91° E	15	B01(4), B02(1), B03(8), B04(2)
Qiantang River				
5. Pan’an County, Zhejiang Province	29.06° N	120.45° E	1	B05(1)
6. Yuqian Town, Lin’an City, Zhejiang Province	30.19° N	119.40° E	6	B06(2), B07(1), B08(2), B09(1)
7. Changhua Town, Lin’an City, Zhejiang Province	30.17° N	119.22° E	10	B08(3), B10(4), B11(1), B12(1), B13(1)
8. Yunti Village, Ningguo City, Anhui Province	30.37° N	119.21° E	11	B05(1), B08(2), B12(1), B14(1), B15(1), B16(4), B17(1)
9. Huangshan City, Anhui Province	29.72° N	118.31° E	1	B05(1)
10. Jiangshan City, Zhejiang Province	28.76° N	118.66° E	1	B18(1)
Yangtze River				
11. Anji County, Zhejiang Province	30.64° N	119.67° E	6	A13(5), A14(1)
12. Ningguo City, Anhui Province	30.62° N	118.95° E	7	A15(1), A16(1), A17(1), A18(1), A19(1), A20(1), A21(1)
13. Shitai County, Anhui Province	30.21° N	117.50° E	10	A22(1), A23(2), A24(1), A25(1), A26(2), A27(1), A28(1), A29(1)
14. Fuliang County, Jiangxi Province	29.52° N	117.28° E	1	C01(1)
15. Yushan County, Jiangxi Province	28.69° N	118.28° E	1	C02(1)
16. Fengxin County, Jiangxi Province	28.71° N	115.38° E	6	C03(1), C04(2), C05(1), C06(1), C07(1)
17. Jing’an County, Jiangxi Province	28.85° N	115.39° E	18	C04(2), C08(13), C09(1), C10(1), C11(1)
18. Tonggu County, Jiangxi Province	28.53° N	114.39° E	9	C06(5), C12(2), C13(1), C14(1)
19. Xiushui County, Jiangxi Province	29.02° N	114.54° E	1	C15(1)
20. Ningdu County, Jiangxi Province	26.46° N	116.00° E	5	C16(3), C17(2)
21. Suichuan County, Jiangxi Province	26.31° N	114.52° E	15	C16(9), C17(2), C18(1), C19(3)
22. Anfu County, Jiangxi Province	27.39° N	114.63° E	1	C16(1)

Lat.—latitude; Long.—longitude; N—number of individuals; number of individuals for each haplotype is in bracket.

**Table 2:** Genetic diversity of *H. chenhsienensis*.

Basin	Number	Number of HaploTypes (*Nh*)	Number of Private Haplotypes (*Nph*)	Haplotype Diversity (*h*)	Nucleotide Diverstiy (*π*)
Ou River	16	11	11	0.9500 ± 0.0364	0.0028 ± 0.0017
Jiao River	1	1	1	-	-
Cao’e River	15	4	4	0.6667 ± 0.0991	0.0007 ± 0.0006
Qiantang River	30	14	14	0.9126 ± 0.0307	0.0024 ± 0.0015
Yangtze River	80	36	36	0.9370 ± 0.0149	0.0371 ± 0.0180
Total	142	66	66	0.9722 ± 0.0054	0.0460 ± 0.0222

**Table 3:** Pairwise comparison Φ_ST_ values among basins (lower diagonal) and associated *p* values (upper diagonal) of *H. chenhsienensis*.

	Ou River	Cao’e River	Qiantang River	Yangtze River
Ou River		**0.000**	**0.000**	**0.000**
Cao’e River	0.986		**0.000**	**0.000**
Qiantang River	0.981	0.367		**0.000**
Yangtze River	0.546	0.510	0.559	

Jiao River was excluded from the analysis because it had only one sample. Significant *p* values (*p* < 0.05) by Bonferroni correction are displayed in bold.

**Table 4:** Neutrality tests for lineages A, B, and C of *H. chenhsienensis*.

Lineage	Tajima’s *D*	Fu’s *Fs*
A	0.320 (*p* = 0.706)	−4.231 (*p* = 0.095)
B	−1.768 (*p* = 0.014)	−9.126 (*p* = 0.000)
C	0.528 (*p* = 0.781)	1.220 (*p* = 0.714)

## Data Availability

The newly generated sequences of mitochondrial *Cytb* gene and genomes in this study are available on GenBank (http://www.ncbi.nlm.nih.gov, accessed on 27 October 2022) with the following accession number: OM647979–OM648044 (*Cytb* sequences), OM743247–OM743251 (*Cytb* sequences) and ON316823–ON316826 (genomes sequences).

## Supplementary Materials

The following supporting information can be downloaded at: https://www.mdpi.com/article/10.3390/life12071024/s1, Figure S1: Time tree of *Huigobio* and its close relatives based on 13 protein-coding genes of mitochondrial genomes; Table S1: Reviews of the genetic diversity of freshwater fishes endemic to southern China revealed by mitochondrial genes; Table S2: Reviews of the phylogeographical patterns of freshwater fishes endemic to southern China revealed by mitochondrial genes; Table S3: Primers and annealing temperature used in PCR amplification of mitochondrial genomes; Table S4: Sampling localities and source of mitochondrial genome for *Huigobio* and outgroup taxa; Table S5: Comparisons among six models of biogeography analyses in ancestral area reconstruction of *H. chenhsienensis*. References [11,13,14,15,16,17,18,26,27,37,65,72,73,74,75,76,77,78,79,80,81,82,83,84,85,86,87,88,89,90,91,92,93,94,95,96,97,98,99,100,101,102,103,104,105,106] are cited in the supplementary materials.
