# Supplementary material for: Phylogeographical Analysis of the Freshwater Gudgeon Huigobio chenhsienensis (Cypriniformes: Gobionidae) in Southern China
Source: Life (Basel). 2022 Jul 9;12(7):1024. doi: 10.3390/life12071024 (PMC9318155; doi:10.3390/life12071024)
Supplement: Supplementary file 1 [file life-12-01024-s001.zip › life-1766717-supplementary.pdf]

Supplementary Materials

Phylogeographical Analysis of the Freshwater Gudgeon *Huigobio chenhsienensis* (Cypriniformes: Gobionidae) in Southern China

Xishu Yang , Xiaomin Ni , and Cuizhang Fu *

Ministry of Education Key Laboratory for Biodiversity Science and Ecological Engineering, Coastal Ecosystems Research Station of the Yangtze River Estuary, Institute of Biodiversity Science and Institute of Eco-Chongming, School of Life Sciences, Fudan University, Shanghai, 200082, China; yangxishu1990@163.com (X.Y.); 18110700109@fudan.edu.cn (X.N.); czfu@fudan.edu.cn (C.F.)

* Correspondence: czfu@fudan.edu.cn

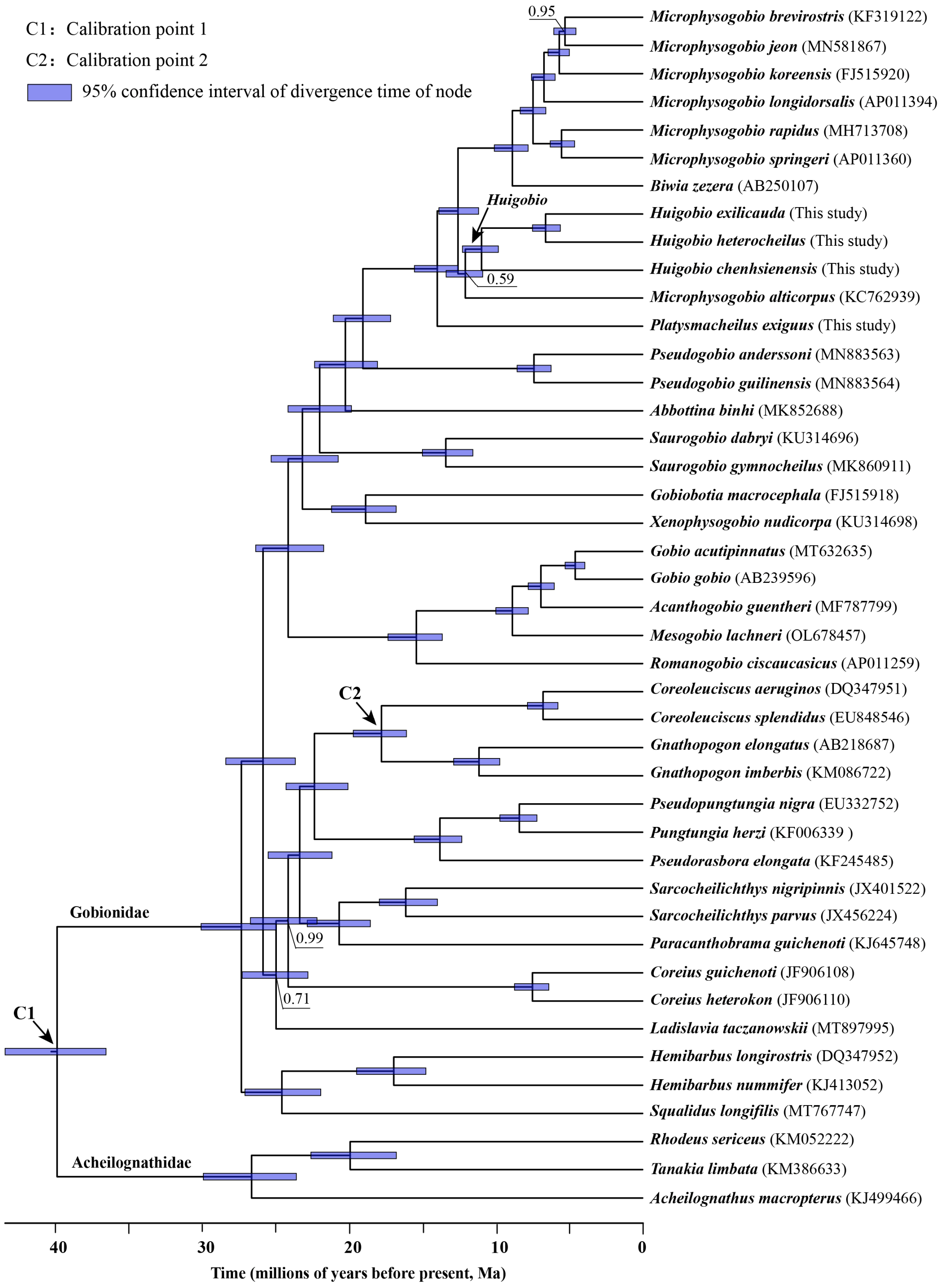

Figure S1. Time tree of *Huigobio* and its close relatives based on 13 protein-coding genes of mitochondrial genomes. The posterior probability of the node is not shown when the value is 1. Data sources of species see Table S4. Genbank accession number within the brackets.

Table S1. Reviews of the genetic diversity of freshwater fishes endemic to southern China revealed by mitochondrial genes

Category	Order	Species	Basin	*h*	π	References
II	Cypriniformes	*Sinibrama macrops*	Qiantang River, Cao'e River	0.9610	0.0044	[72]
		Schizothorax nukiangensis	Nujiang River	0.9650	0.0024	[73]
		Acrossocheilus longipinnis	Pearl River	0.6859	0.0036	[74]
IV	Cypriniformes	*Aphyocypris normalis*	Pearl River, Jianjiang River, Moyang River, Beilun River, Nandu River, Wanquan River, Zhubijang River, Lingshui River, Changhua River	1.0000	0.0149	[14]
		Onychostoma lepturum	Nandu River, Wanquan River, Lingshui River, Changhua River	0.9420	0.0229	[17]
		Tanichthys albonubes	Luo River, Pearl River, Beilun River, Red River, Changhua River	0.9700	0.0516	[75]
		Poropuntius huangchuchieni	Mekong River, Red River, Nujiang River	0.9856	0.0287	[11]
		Garra orientalis	Min River, Jiulong River, Pearl River, Moyang River, Wanquan River, Nandu River, Changhua River	0.9810	0.0074	[18]
		Opsariichthys hainanensis	Tan River, Moyang River, Jianjiang River, Nanliujiang River, Red River, Longwei River, Longshou River, Lingshui River, Wanquan River, Nandu River, Changhua River	0.9460	0.0149	[76]
	Perciformes	*Coreoperca whiteheadi*	Yangtze River, Qiantang River, Moyang River, Pearl River	0.8048	0.0601	[15]
	Siluriformes	*Glyptothorax fokiensis*	Yangtze River, Min River,Jiulong River, Hanjiang River, Pearl River, Moyang River	0.9920	0.0406	[13]
		Glyptothorax hainanensis	Jianjiang River, Lianjiang River, Nanliujiang River, Wanquan River	1.0000	0.0188	[13]

Table S2. Reviews of the phylogeographical patterns of freshwater fishes endemic to southern China revealed by mitochondrial genes

Pattern	Order	Species	References
I	Cypriniformes	*Hemibagrus guttatus*	[65]
		Aphyocypris normalis	[14]
		Tanichthys albonubes	[75]
		Poropuntius huangchuchieni	[11]
	Perciformes	*Coreoperca whiteheadi*	[15]
	Siluriformes	*Glyptothorax fokiensis*	[13]
		Glyptothorax hainanensis	[13]
II	Cypriniformes	*Schizothorax nukiangensis*	[73]
		Acrossocheilus longipinnis	[74]
		Onychostoma lepturum	[17]
		Opsariichthys hainanensis	[76]
	Siluriformes	*Hemibagrus macropterus*	[16]
V	Cypriniformes	*Garra orientalis*	[18]

Table S3. Primers and annealing temperature used in PCR amplification of mitochondrial genomes

Fragment	Gene	Site	Name of primer	Squence (5'-3')	Source	Annealing temperature
1	12S-16S	72-1479	T-Gob12SF	AAGGCATGGTCCYGACCTTA	[27]	55.4 °C
			CGob16sR	TTCGGTAGGTCTRTCACTTC	[27]	
2	Val-ND1	1066-2870	CGobValF	ACACCGAGAAGACATCCA	[77]	54.3 °C
			CGobLeuR1	GGGAAGAGGAYTTGAACC	[27]	
3	16S-ND2	2572-4072	CGobND1F	GCAGCCGCTATTAAGGGTT	[27]	52.3 °C
			CYMPHND1R	TTTATTATGAGAATGTTGGC	This study	
4	Ile-COI	3870-5570	T-SD-IleF	GCCCAAGGACCACTTTGATAG	[27]	52.5 °C
			CGobCo1R2	CCAAATACRAGATARAGGGT	[77]	
5	Asn-Ser	5312-7109	LY-AsnF	AGCGAGCATCCATCTACTT	[27]	54.6 °C
			CARSerR	GGTYATGTGACTGGCTTGA	[27]	
6	COI-ATP6	6958-8866	TGobCOIF	TGAGAAGCCTTYGCCGCYAAACG	[27]	52.4 °C
			TGobATP6R	GCTTGGTGTGCCATTARACGTTTTCTTG	This study	
7	ATP6-Arg	8528-10066	ZXSATP6F	AATCAACCAACMGTAGCYCT	This study	53.3 °C
			CGobArgR	CTGAGYCGAAATCAGAGG	[27]	
8	Gly-ND4	9665-11020	CGobGlyF	CTTCCAATYATTTAGYCTTGG	[77]	53.5 °C
			NMIND4R	TGCAACAGGGGCYTCTAC	This study	
9	ND4L-Leu	10660-12010	TGobND4FS	TAGCCAGCCAAAAYCACAT	This study	54.5 °C
			CGobLeuR	TGGAYTTGCACCAAGAGT	[27]	

Table S3 (*continued*)

Fragment	Gene	Site	Name of primer	Squence (5'-3')	Source	Annealing temperature
10	His-ND5	11822-13375	CGobSerF1-M	ACYCACCRAGGAAGGACA	This study	50.0 °C
			CYND5R	ATAATTGAYCCYGAGCA	This study	
11	ND5-CYTB	12949-14471	TGobND5F	GTAGCTTTCTCRACATC	This study	50.5 °C
			NMICYTBR	CGTAGGCTTGCCATTATT	This study	
12	ND6-Pro	14322-15680	CGluF	TCTTGCTCGGAYTTTAACCGAG	[26]	58.0 °C
			CThrR	CCTCCGATCTTCGGATTACAAG	[26]	
13	Thr-12S	15566-217	HemDloopF	AAAGCATCGGTCTTGTAATC	[27]	53.0 °C
			HemDloopR	CTTGGCTAGGCGTCTTGG	[27]	

Table S4. Sampling localities and source of mitochondrial genome for *Huigobio* and outgroup taxa

Species	Locality	Genbank accession number	References
Huigobio			
Huigobio chenhsienensis	Xinchang, Zhejiang Province, China	ON316823	This study
Huigobio exilicauda	Shixing, Guangdong Province, China	ON316824	This study
Huigobio heterocheilus	Yanling, Hunan Province, China	ON316825	This study
Microphysogobio			
Microphysogobio jeon	Buyeo-gun, South Korea	MN581867	[78]
Microphysogobio springeri (=*Abbottina springeri*=*Biwia springeri)*		AP011360	[37]
Microphysogobio brevirostris		KF319122	[79]
Microphysogobio koreensis	South Korea	FJ515920	[80]
Microphysogobio longidorsalis	South Korea	AP011394	[37]
Microphysogobio rapidus	South Korea	MH713708	[81]
Microphysogobio alticorpus		KC762939	[82]
Biwia			
Biwia zezera	Gifu, Japan	AB250107	[83]
Platysmacheilus			
Platysmacheilus exiguus	Yangsuo, Guangxi Zhuang Autonomous Region, China	ON316826	This study
Gobiobotia			
Gobiobotia macrocephala	South Korea	FJ515918	[84]
Xenophysogobio			
Xenophysogobio nudicorpa	Jiangjin,Chongqing Municipality, China	KU314698	[85]

Table S4 (*continued*)

Species	Locality	Genbank accession number	References
Hemibarbus			
Hemibarbus nummifer (=*Belligobio nummifer*)	Wuyishan, Fujian Province, China	KJ413052	[86]
Hemibarbus longirostris	Ganwon-do, South Korea	DQ347952	[87]
Squalidus			
Squalidus longifilis	Fengcheng, Liaoning Province, China	MT767747	[27]
Abbottina			
Abbottina binhi	Pingguo, Guangxi Zhuang Autonomous Region, China	MK852688	[88]
Pseudogobio			
Pseudogobio anderssoni	Anyi, Jiangxi Province, China	MN883563	[89]
Pseudogobio guilinensis	Pingle, Guangxi Zhuang Autonomous Region, China	MN883564	[89]
Saurogobio			
Saurogobio dabryi	Xiushui, Jiangxi Province, China	KU314696	[85]
Saurogobio gymnocheilus	Hengdong, Hunan Province, China	MK860911	[90]
Gobio			
Gobio acutipinnatus	Bu'erjin, Xinjiang Uygur Autonomous Region, China	MT632635	[91]
Gobio gobio	Plana	AB239596	[92]
Mesogobio			
Mesogobio lachneri	Linjiang, Jilin Province, China	OL678457	unpublished (from Genbank)
Acanthogobio			
Acanthogobio guentheri		MF787799	unpublished (from Genbank)
Romanogobio			
Romanogobio ciscaucasicus		AP011259	unpublished (from Genbank)

Table S4 (*continued*)

Species	Locality	Genbank accession number	References
Gnathopogon			
Gnathopogon elongatus	Japan	AB218687	[92]
Gnathopogon imberbis (=*Gnathopogon polytaenia*)		KM086722	[93]
Coreoleuciscus			
Coreoleuciscus splendidus	South Korea	EU848546	[94]
*Coreoleuciscus aeruginos**	South Korea	DQ347951	[94]
Pseudorasbora			
Pseudorasbora elongata	Wuyuan, Jiangxi Province, China	KF245485	[95]
Pungtungia			
Pungtungia herzi	South Korea	KF006339	[96]
Pseudopungtungia			
Pseudopungtungia nigra	South Korea	EU332752	[97]
Sarcocheilichthys			
Sarcocheilichthys parvus	Yongjia, Zhejiang Province, China	JX456224	[98]
Sarcocheilichthys nigripinnis	Kaihua, Zhejiang Province, China	JX401522	[99]
Paracanthobrama			
Paracanthobrama guichenoti		KJ645748	[100]
Coreius			
Coreius guichenoti	Banan, Chongqing Municipality, China	JF906108	[101]
Coreius heterokon	Banan, Chongqing Municipality, China	JF906110	[101]
Ladislavia			
Ladislavia taczanowskii	Kuandian, Liaoning Province, China	MT897995	[102]

Table S4 (*continued*)

Species	Locality	Genbank accession number	References
Acheilognathus			
Acheilognathus macropterus	Wuhan, Hubei Province, China	KJ499466	[103]
Rhodeus			
Rhodeus sericeus	Xiaoxing'anling, Heilongjiang Province, China	KM052222	[104]
Tanakia			
Tanakia limbata	Okayama, Japan	KM386633	[105]

*The scientific name in Genbank is *Coreoleuciscus splendidus* that it is revised as *Coreoleuciscus aeruginos* by Song and Bang [106].

Table S5. Comparisons among six models of biogeography analyses in ancestral area reconstruction of *H. chenhsienensis*

Model	LnL	Parameter			Weight	
		d	*e*	*j*	AIC_wt	AICc_wt
DEC	-30.11	1.80E-02	1.00E-12	0.00	4.60E-03	5.07E-03
DEC+J	-25.77	1.00E-12	1.00E-12	5.48E-03	1.29E-01	1.29E-01
DIVALIKE	-32.23	3.08E-02	8.47E-03	0.00	5.51E-04	6.08E-04
**DIVALIKE+J**	**-23.87**	**1.00E-12**	**1.00E-12**	**5.40E-03**	**8.65E-01**	**8.65E-01**
BAYAREALIKE	-50.07	4.77E-02	4.01E-01	0.00	9.80E-12	1.08E-11
BAYAREALIKE+J	-31.56	1.00E-07	8.35E-02	8.72E-03	3.97E-04	3.97E-04

The optimal model is highlighted in bold based on LnL and weight values.
